# Exploring Plant Extracts as a Novel Frontier in Antimicrobial Innovation: Plant Extracts and Antimicrobials

**DOI:** 10.3390/microorganisms12030483

**Published:** 2024-02-27

**Authors:** Roberto Venanzoni, Giancarlo Angeles Flores, Paola Angelini

**Affiliations:** 1 Department of Chemistry, Biology and Biotechnology, University of Perugia, 06122 Perugia, Italy; roberto.venanzoni@unipg.it; 2 Botanic Garden “Giardino dei Semplici”, Department of Pharmacy, “Gabriele d’Annunzio” University, 66100 Chieti, Italy; giancarlo.angelesflores@unich.it

The scientific exploration presented in this Special Issue offers a comprehensive overview of recent advancements in the realm of plant-derived antimicrobials. Notable findings have emerged from this collection, such as the revelation of the antifungal properties of a hydroalcoholic extract of the mushroom *Hypsizygus marmoreus* (Peck) H.E. Bigelow against dermatophytes—an indication of the largely unexplored potential within our natural resources, as demonstrated by Angelini et al. [1]. However, this edition stands out as a testament to the wide array of plant extracts and compounds that are effective at addressing microbial threats.

The topics covered in this issue span a wide spectrum, including the production of plant compounds with antimicrobial properties, their industrial applications, and how to scale them up. 

Canly et al. [2] have conducted a study that elucidates the potent antimicrobial properties of an ethanol extract derived from *Origanum onites* L. (OOEt). This extract has demonstrated significant efficacy against a diverse spectrum of pathogenic microorganisms, including both bacterial and fungal strains. Notably, in certain instances, the antimicrobial effectiveness of OOEt surpassed that of synthetic antibiotics, underscoring its promising role as a natural alternative in combating infections.

In a separate study, Moreau et al. [3] investigated the antifungal properties of various components of hops (*Humulus lupulus* L.) against *Venturia inaequalis* (Cooke) G. Winter, the causative agent of apple scab. Extracts from hops, including leaves and stems, and some of their specific metabolites, were tested in vitro. Two types of extracts, a crude hydro-ethanolic extract and a dichloromethane sub-extract, were used to evaluate their impact on the spore germination of two strains of *V. inaequalis*, each with distinct sensitivities to triazole fungicides. The extracts from the cones, leaves, and stems of hops showed inhibitory effects on both strains, while their rhizomes exhibited no activity. The most effective extract underwent fractionation into seven fractions using preparative HPLC. These fractions were then screened for antifungal properties, with a focus on the chalcone xanthohumol, which was isolated from the most potent fraction. In conclusion, the findings suggest that hop extracts and xanthohumol hold promise as botanical agents for controlling the phytopathogen *V. inaequalis*.

Similarly, Al Otibi et al. [4] explored the antifungal characteristics of *Tamarix aphylla* (L.) H.Karst. sourced from the Saudi desert, specifically targeting five filamentous fungal species: *Macrophomina phaseolina* (Tassi) Goid., *Curvularia spicifera* (Bainier) Boedijn, *Fusarium verticilliodes* (Sacc.) Nirenberg, *F. solani* (Mart.) Sacc., and *F. monoliforme* J. Sheld. The fungicidal potential of *T. aphylla* against these species had not been previously assessed, underscoring the novelty of this research.

Advancing the field, Wang et al. [5] introduced an innovative approach that combines tea polyphenols, gallic acid, and cinnamon essential oil to create a complex natural extract microemulsion system (NM). This system demonstrates robust antibacterial and antioxidant activities while ensuring its own stability and causing minimal irritation. This study’s implications are significant for the potential use of plant extracts as natural preservatives in both the food and cosmetics industries.

Simultaneously, Sateriale et al. [6] conducted a thorough investigation into the antibacterial properties of essential oils from thyme (*Thymus vulgaris* L.) and cloves [*Syzygium aromaticum* (L.) Merr. & L.M.Perry] against a range of Gram-positive and Gram-negative foodborne pathogenic bacteria. The chemical composition of their essential oils was meticulously determined through gas chromatography–mass spectrometry analysis. Using qualitative in vitro antimicrobial assays, such as the agar well diffusion method and disk-volatilization method, the efficacy of these essential oils was evaluated individually, in binary combinations, and in both liquid and vapor phases against *Staphylococcus aureus* and *Escherichia coli* food isolates. Their findings strongly support the potential of these essential oils as promising sources for the development of new, broad-spectrum, and environmentally friendly food preservatives.

Abdelhamed et al. [7] provide a thorough examination of the antimicrobial properties inherent to five essential oils commonly used in the Mediterranean region, with a specific focus on their effectiveness at addressing acne vulgaris. The main goal of their study was to clarify the mode of action of the most potent essential oil, investigating its impact on bacterial cell membranes and intracellular components. Additionally, their research aimed to create a pharmaceutical formulation of the essential oil with the highest antimicrobial efficacy. Thyme essential oil, identified as the most effective through in vitro antibacterial tests against acne-causing bacteria, was subsequently formulated into a nanoemulsion. 

In a related study, Milia et al. [8] addressed the challenge of countering a microbiota which contains carbohydrate-fermenting *Streptococcus mutans* and species of various genera that may contribute to dental caries on tooth surfaces. Their paper highlights plant-derived substances as potential alternatives to conventional antiseptics and antibiotics. The use of *Pistacia lentiscus* L. polyphenols in preventing and/or reversing intraoral dysbiosis is strongly supported by their inhibition of periodontal pathogens and *Candida albicans*, coupled with their antioxidant activity and reduced inflammatory responses. This extract appears notably more effective than other derivatives of *Pistacia lentiscus* L. compounds, emphasizing the need to refine our extraction techniques [9]. The integration of plant extracts into various sectors, including food, beverages, supplements, and cosmetics, has been a central focus of research, promising a comprehensive incorporation of nature’s gifts into our daily lives [10].

As we reflect on the strides made in the field, it is necessary to acknowledge the gaps that persist in our knowledge. While this Special Issue has been instrumental in unraveling the potential of plant extracts, there is still much uncharted territory. Future research should delve deeper into understanding the intricacies of microbial pathogenicity and other infectious diseases in order to refine our arsenal against these evolving threats [11]. Moreover, exploring sustainable practices for the large-scale production and dissemination of these plant-derived antimicrobials is a pathway that merits dedicated attention [12].

In conclusion, “Plant Extracts and Antimicrobials” is not just a collection of scientific contributions but a manifesto for embracing nature’s potential in our ongoing battle against microbial challenges. The seeds planted by this Special Issue are destined to grow into a lush garden of knowledge, yielding solutions that transcend conventional boundaries. As we turn the pages of this issue, let us look to the future with anticipation, where the synergy between science and nature will continue to flourish, providing sustainable solutions to global health challenges.
